# The Interplay between *Helicobacter pylori* and Gut Microbiota in Non-Gastrointestinal Disorders: A Special Focus on Atherosclerosis

**DOI:** 10.3390/ijms242417520

**Published:** 2023-12-15

**Authors:** Marcello Candelli, Laura Franza, Rossella Cianci, Giulia Pignataro, Giuseppe Merra, Andrea Piccioni, Veronica Ojetti, Antonio Gasbarrini, Francesco Franceschi

**Affiliations:** 1 Emergency, Anesthesiological and Reanimation Sciences Department, Fondazione Policlinico Universitario A. Gemelli—IRCCS of Rome, 00168 Rome, Italy; laura.franza@policlinicogemelli.it (L.F.); giulia.pignataro@policlinicogemelli.it (G.P.); andrea.piccioni@policlinicogemelli.it (A.P.); veronica.ojetti@policlinicogemelli.it (V.O.); francesco.franceschi@policlinicogemelli.it (F.F.); 2 Department of Translational Medicine and Surgery, Catholic University, Fondazione Policlinico Universitario A. Gemelli—IRCCS, 00168 Rome, Italy; rossella.cianci@unicatt.it; 3 Biomedicine and Prevention Department, Section of Clinical Nutrition and Nutrigenomics, Facoltà di Medicina e Chirurgia, Università degli Studi di Roma Tor Vergata, 00133 Rome, Italy; giuseppe.merra@uniroma2.it; 4 Medical, Abdominal Surgery and Endocrine-Metabolic Science Department, Fondazione Policlinico Universitario A. Gemelli—IRCCS of Rome, 00168 Rome, Italy; antonio.gasbarrini@policlinicogemelli.it

**Keywords:** atherosclerosis, *H. pylori*, gut microbiota, Cag-A, inflammation

## Abstract

The discovery of Helicobacter pylori (*H. pylori*) in the early 1980s by Nobel Prize winners in medicine Robin Warren and Barry Marshall led to a revolution in physiopathology and consequently in the treatment of peptic ulcer disease. Subsequently, *H. pylori* has also been linked to non-gastrointestinal diseases, such as autoimmune thrombocytopenia, acne rosacea, and Raynaud’s syndrome. In addition, several studies have shown an association with cardiovascular disease and atherosclerosis. Our narrative review aims to investigate the connection between *H. pylori* infection, gut microbiota, and extra-gastric diseases, with a particular emphasis on atherosclerosis. We conducted an extensive search on PubMed, Google Scholar, and Scopus, using the keywords “*H. pylori*”, “dysbiosis”, “microbiota”, “atherosclerosis”, “cardiovascular disease” in the last ten years. Atherosclerosis is a complex condition in which the arteries thicken or harden due to plaque deposits in the inner lining of an artery and is associated with several cardiovascular diseases. Recent research has highlighted the role of the microbiota in the pathogenesis of this group of diseases. *H. pylori* is able to both directly influence the onset of atherosclerosis and negatively modulate the microbiota. *H. pylori* is an important factor in promoting atherosclerosis. Progress is being made in understanding the underlying mechanisms, which could open the way to interesting new therapeutic perspectives.

## 1. Methodology

In the present narrative review, we want to specifically focus on the interaction between gut microbiota (GM) and *H. pylori* in extragastric diseases, and in particular atherosclerosis. To this purpose, we have conducted a literature search on different databases (PubMed, Google Scholar, Scopus), using the key terms “*H. pylori*”, “dysbiosis”, “microbiota”, “atherosclerosis”, “cardiovascular disease”. We focused our research on in vitro, animal, and human study. For the critical analysis of our review, we examined the papers published in the last ten years (2013–2023).

## 2. Cardiovascular Disease: Risk Factors

Cardiovascular diseases (CVDs) are the leading cause of mortality and morbidity worldwide. According to the World Health Organization (WHO), approximately 18 million people die each year from CVDs or their complications [1]. Over the last seventy years, the incidence of CVDs has increased, replacing communicable diseases as the first cause of death in the general population [2]. Several studies have investigated the risk factors for this group of diseases [3,4,5]. While it is not possible to change any of these factors, there are others that can be the target of interventions, such us smoking and diet [6,7]. Diet has been associated with CVDs in many studies. For example, a 2020 study by Xu et al. found that individuals with a healthier diet had a lower risk of developing CVDs. The Mediterranean diet has been associated with a protective effect against the development of CVDs [8], while the Western diet appears to instead promote this group of disorders [9,10,11]. While the first studies conducted on this topic suggested a direct link between high fat consumption and atherosclerosis and lipid accumulation in general, the relationship between cardiovascular health and diet seems to be more complicated [11]. Diet affects mitochondrial function, but its influence on inflammation also seems to play a significant role. In mice, for example, it has been observed that a Western diet is able to single-handedly alter inflammatory pathways and innate immune responses and promotes the proliferation of myeloid progenitor cells [12]. Some components of the Western diet, such as fatty acids and triglycerides, are directly associated with inflammatory diseases. It has been observed, for instance, that these elements can trigger inflammation, for instance, through the activation of the NLP3 inflammasome and Toll-like receptor (TLR)-4 [13,14,15]. The effects of diet on the immune system are also strongly influenced by the gut microbiota (GM).

### 2.1. Cardiovascular Disease: The Role of Microbiota

The GM consists of all the microorganisms that live in our gut and is very dynamic and complex [16]. It exerts several functions that can be broadly divided into metabolic, mechanical, and immunological. The latter is particularly relevant to our topic, as a close relationship has been observed between CVDs and inflammation, which is especially significant in the case of atherosclerosis [17]. GM composition is associated with the activation of various inflammatory processes, which in turn play a key role in promoting CVDs. As described by Witkowski et al. [18], metabolites and bacterial products of GM are able to activate the immune system through specific pathways, which determine specific effects. In addition, the authors described a general state of dysbiosis in patients with CVDs. Dysbiosis is a state of imbalance in which a relative abundance of some microbial species compared to others is observed and is associated with a loss of microbial diversity [19]. Dysbiosis has different effects on the homeostasis of the organism and contributes to the development of CVDs through different mechanisms. One of the most important mechanisms by which the microbiota can promote microinflammation and subsequently CVDs is the production of metabolites that interact with the host immune system. For example, Lipopolysaccharide (LPS), which makes up the surface glycolipids of Gram-negative bacteria, is produced by GM and leads to mild inflammation via activation of various TLRs, particularly TLR-9, -2, and -4, which, as mentioned earlier, are key mediators in fatty acid-induced inflammation. Proline-rich extensin-like receptor kinases (PERK5) are instead activated by trimethylamine N-oxide (TMAO), which is derived from the product of phosphatidylcholine, found in meat and dairy products, and has been associated with cardio-renal axis changes [20]. The gut-kidney-heart axis is also affected by P-cresol sulfate and indoxyl sulfate, which are uremic toxins whose metabolism can be modulated by the composition of GM [21]. In this case, activation of the aryl hydrocarbon receptor (AHR) expressed by various immune cells leads to an increase in reactive oxygen species (ROS) and fibrosis, as well as impaired cardiac and renal function [22]. Phenylacetylglutamine (PAG) is another GM metabolite, which can influence both kidney and CV health: it is metabolized from phenylamine and determines the activation of adrenergic receptors, promoting high blood pressure, endothelial dysfunction, and thrombosis [23]. However, GM may also exert a protective function through the metabolism of bile acids, modulating the metabolism of lipids and carbohydrates, and reducing inflammation by regulating the activation of Takeda G-protein-coupled receptor (TGR)-5, liver X receptor (LXR), and farnesol X receptor (FXR) [24,25,26]. Other useful metabolites produced by GM are short-chain fatty acids (SCFAs). GM metabolizes acetate, propionate, and butyrate, which can modulate G protein-coupled receptors (GPR)-41 and -43 and olfactory receptor (Olfr)-78. GPR-41 and -43 appear to have a protective effect against inflammation and obesity [27], while Olfr-78 directly affects intestinal inflammation [28]. The metabolites described above can also affect the immune system via the so-called “leaky gut”. In this condition, the intestinal barrier is disrupted, allowing bacteria and their products to enter the bloodstream. It has been observed that leaky gut may be associated with an increased risk of CVDs. In particular, those associated with dysbiosis have been linked to the activation of inflammation, primarily through the interleukin (IL)-1β pathway [18]. Some microorganisms of the intestinal microbiota, particularly pathogens, may also directly affect cardiovascular health. In a study by Li et al. [29], for instance, the presence of *C. pneumoniae*, *P. gingivalis*, *H. pylori*, *Cytomegalovirus (CMV)*, *Epstein–Barr virus (EBV)*, *human immunodeficiency virus (HIV)*, *herpes simplex virus-1 (HSV-1)*, *HSV-2* and *hepatitis C virus (HCV)*, were all associated with an increased risk of CVDs.

### 2.2. The Case of Atherosclerosis

Atherosclerosis is a complex disease characterized by the presence of plaques in the arteries composed of cholesterol, fat, and blood cells [30]. While atherosclerosis has historically been considered primarily a consequence of lifestyle and diet, other hypotheses have been formulated, and some authors suggest that atherosclerosis may be considered an inflammatory disorder. Low-density lipoprotein (LDL) cholesterol, hypertension, and smoking are among the most important risk factors for atherosclerosis, but inflammation is a prerequisite for the development of the disease. Inflammation plays an important role in endothelial dysfunction. When the endothelium is damaged, it begins to express several receptors (e.g., vascular cell adhesion molecule 1-VCAM-1-, Intercellular Adhesion Molecule 1-ICAM-1-, E- and P-selectin), all of which are capable of attracting various immune cells that further promote the inflammatory process. The cellular components present at the site of action promote the accumulation of oxidized LDL, which increases the size of the plaque [31]. As the process progresses, macrophages invade the vessel and promote plaque rupture by degrading collagen and expressing matrix metalloproteinases (MMPs) [32]. Another aspect that must be considered is the role of different specific inflammatory pathways in the development of atherosclerosis. Whereas the Janus kinase (JAK)-signal transducer of activators of transcription (STAT) pathway (JAK-STAT) and nuclear factor kappa-light-chain-enhancer of activated B cells (NF-κB) are usually activated by cytokine cascades, TLR-4, another major component of inflammation leading to atherosclerosis, is primarily activated by bacterial byproducts, more specifically by LPS [33]. The discovery of the importance of TLR-4 in the pathogenesis of atherosclerosis prompted research on the role of infections and the gut microbiota in this disease. As mentioned above, several pathogens have been linked to the development of CVDs and atherosclerosis [29]. Interestingly, one of the bacteria involved in the pathogenesis of atherosclerosis, *Porphyromonas gingivalis*, which is associated with the development of periodontal disease, appears to induce activation of the NF-κB inflammatory pathway, in addition to TLR-4 [34]. Another finding confirming the importance of infection is the observation that bacterial DNA may be present in the atherosclerotic plaque [35]. *C. pneumoniae* and *H. pylori* are among the most prevalent.

However, it appears that the microbiota in general plays an important role in the development of atherosclerosis, through three different mechanisms, as shown by Jonsson et al. [35]. The first mechanism is the role of GM in the metabolism of cholesterol and lipids, and the second is the production of specific metabolites that can promote or inhibit the atherosclerotic process. Finally, the composition of GM itself has been linked to the development of atherosclerosis. Several studies have shown that the presence of Proteobacteria, Actinobacteria, and Firmicutes is associated with an increased risk of atherosclerosis, whereas Bacteroidetes are more abundant in individuals who do not have atherosclerosis [36]. Overall, it appears that GM composition can promote the onset and progression of atherosclerosis through both metabolic and immunological pathways, in which *H. pylori* plays a significant role. These mechanisms are also involved in the onset of other extra-intestinal disorders associated with this infection, as discussed below.

## 3. *H. pylori*: Not Only Gastritis

*H. pylori* is a Gram-negative, microaerophilic, spiral-shaped bacterium first discovered in 1982 by Barry J. Marshall and Robin Warren, who subsequently understood its role in the pathogenesis of gastritis and peptic ulcers [37]. *H. pylori* is usually found in the stomach, and although it is considered an extracellular bacterium, it can be localized under the mucus layer of the gastric epithelium, which allows it to survive in the extremely hostile environment of the stomach [38]. *H. pylori* uses several strategies to invade the gastric mucosa. A key component for mucosal colonization is the use of its flagella, which allow it to colonize the epithelium, as it has been observed that loss of flagellar function prevents colonization [39]. Another key component in adhesion and colonization is chemotaxis. T1pA, B, C, D, and CheA kinase are among the various receptors that allow bacteria to individualize the areas where urea, histidine, glutamine, glycine, and arginine have higher concentrations, while avoiding chemo repellent substances (e.g., bile acids) [40]. *H. pylori* also expresses adhesion molecules such as blood group antigen-binding adhesin A (BabA), which enable it to bind to Lewis H-1 antigens [41]. Finally, *H. pylori* can also bind to cell adhesion molecules related to carcinoembryonic antigen (CEACAM) 1, 3, 5, and 6 via the HopQ protein, which has also been associated with differences in virulence [42]. The classic manifestation of *H. pylori* is gastric disease, especially gastritis and peptic ulcer [43]. Patients with *H. pylori* infection also have a high incidence of dyspepsia, anemia, idiopathic thrombocytopenic purpura, and gastric mucosa-associated lymphoid tissue (MALT) lymphoma and gastric carcinoma [43,44]. *H. pylori* has been identified as one of the major factors in the development of gastric carcinoma, both through inflammation, acting particularly on the Th17 pathway, and hypergastrinemia [45]. Interestingly, however, it also appears to have a protective effect in esophageal cancer, although the mechanisms are not entirely clear [46]. Although the most common pathological manifestation of *H. pylori* is peptic disease, this does not mean that it cannot affect or even determine disorders in other systems and organs [47]. *H. pylori* has indeed been linked to respiratory, endocrine, cardiovascular, dermatological, neurological, gastrointestinal disorders [48,49]. In respiratory diseases, for example, *H. pylori* can have positive or negative effects. *H. pylori* usually promotes a Th1 and Th17 response in the immune system, while inhibiting the Th2 response; the latter has been associated with the development of allergic diseases [50]. In infants, for example, it has been observed that early exposure to *H. pylori* has a protective effect on the development of asthma [51]. However, other authors have observed that adults infected with *H. pylori* have an increased risk of asthma [52]. *H. pylori* infection has also been studied in the context of chronic obstructive pulmonary disease. In this case, infection appears to be associated with worse disease status at baseline, although there are no statistically significant differences in disease progression [53]. Finally, infection also appears to affect the progression of lung cancer, particularly non-small cell lung cancer. In a study by Oster et al. [54], infection with *H. pylori* was found to be associated with poorer treatment outcomes, particularly in patients undergoing immunotherapy.

As mentioned earlier, *H. pylori* has shown a protective effect on esophageal cancer, but it also has other effects on the gastrointestinal tract. For example, *H. pylori* has been observed to be associated with nonalcoholic fatty liver disease (NAFDL) [55]. The hypothesis is that *H. pylori* promotes inflammation, which is associated with metabolic syndrome and obesity [56]. Another interesting association that has been observed is that between *H. pylori* infection and HCV. It has been noted that the prevalence of *H. pylori* is higher in HCV patients than in the general population, and Okushin et al. [56] have observed that the infection is associated with hepatocellular carcinoma (HCC), even though the nature of the association is not yet clear. The effects of *H. pylori* on the gastrointestinal system are also mediated by the microbiota, which is more diverse in infected individuals. Although the implications of this observation are not clear, chronic low grade inflammation promoted by *H. pylori* is thought to promote host resilience to changes in the composition of GM and to gastrointestinal disease [57].

In a study by Lolekha et al. [58], eradication of *H. pylori* was found to improve motor functions in patients with Parkinson’s disease. In Alzheimer’s disease, *H. pylori* has been associated with the acceleration of disease progression [59,60]. Another disease in which *H. pylori* appears to promote development and progression is Guillain–Barré syndrome (GBS). GBS is a complex disease characterized by acute ascending paralytic neuropathy. Patients usually develop the disease after an infection, usually an upper respiratory tract infection [61]. Dardiotis et al. [62] found that *H. pylori* antibodies in serum and cerebrospinal fluid were significantly more abundant in patients with GBS than in the general population. In contrast, the role of *H. pylori* in multiple sclerosis is controversial. Several authors have observed that the prevalence of *H. pylori* is lower in patients with multiple sclerosis than in the general population. It has been hypothesized that early infection in childhood may help prevent the development of autoimmune diseases (“hygiene hypothesis”) [63,64]. However, a meta-analysis by Arjmandi et al. [65], found that active *H. pylori* infection may promote the development of multiple sclerosis. *H. pylori* infection also appears to be linked to a more common ailment in the general population, headaches. The association has been known for over twenty years [66] and in a recent study by Bawand et al. [67] it has even been observed that detection and eradication of the infection is an effective treatment for patients suffering from migraine.

**Figure 1:**
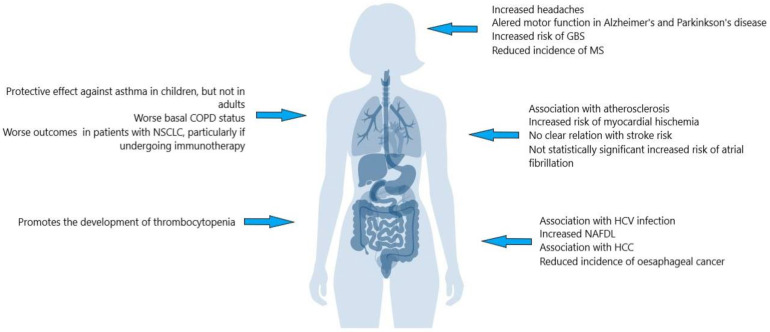
Extra gastric manifestations of *H. pylori.* COPD: chronic obstructive pulmonary disease; GBS: Guillain–Barré syndrome; HCC: hepatocellular carcinoma; HIV: human immunodeficiency virus; MS: multiple sclerosis; NAFDL: non-alcoholic liver disease; NSCLC: non-small cell lung carcinoma.

The links between *H. pylori* and cardiovascular disease, especially atherosclerosis, are discussed in the following section. In Figure 1 a short summary of the extra gastric manifestations of *H. pylori*.

## 4. *H. pylori* and Atherosclerosis

The first reports of a link between atherosclerosis and *H. pylori* date from the mid-1990s. While there was no clear explanation of the underlying mechanisms at that time, some authors began to suggest that the inflammation triggered by the infection might be the main player in this relationship [68]. *H. pylori* can cause an inflammatory response via several pathways. LPS is a component of the outer bacterial membrane of Gram-negative bacteria and activates a Th1 response. However, in *H. pylori* infection, it has been observed that both a Th1 and a Th2 immune response occur. The latter is likely activated by the dendritic cell-specific intercellular adhesion molecule-3-grabbing non-integrin (DCSIGN) lectin pathway, which allows *H. pylori* to induce low-grade inflammation that is less damaging to the gastric mucosa but facilitates colonization [69]. The Th1 immune response to *H. pylori* is also mediated by other components of the bacterium, such as neutrophil-activating protein (HP-NAP). These monomers are released after autolysis and directly activate monocytes and neutrophil granulocytes, which promote the release of IL-8, macrophage inflammatory protein (MIP)-1α, and MIP-1. Monocytes differentiate into dendritic cells (DCs) and begin to produce IL-12 and IL-23. Developing inflammation stimulates mast cells, which begin to produce IL-6 and TNF-α and promote differentiation of T lymphocytes toward Th1 patterns [70]. Vacuolating cytotoxin (VacA) is another key component in modulating the immune response to *H. pylori* infection. On the one hand, it promotes the production of cytokines such as TNF-α, MIP-1α, IL-1, IL-6, IL-10, and IL-13 by activating mast cells; at the same time, it inhibits the proliferation and differentiation of T lymphocytes [71]. In the following paragraphs we will discuss the most peculiar mechanisms through which *H. pylori* and atherosclerosis are linked.

### 4.1. Cytotoxin-Associated Gene Antigen and Atherosclerosis

Cytotoxin-associated gene antigen (CagA) is an important virulence factor for *H. pylori*. This protein is carried into host cells by the bacterial type IV secretion system where it interacts with the host cellular regulation pathway [72].

CagA is able to restrict autophagy in host cells and instead promotes the production of cytokines, specifically IL-1α, IL-8, and IL-18. The pathway by which CagA is able to exert these effects is through the activation of c-Met, which activates the PI3K/AKT/mTOR pathway. The inhibition of autophagy is also accompanied by the accumulation of the sequestrosome (SQSTM)-1 protein, which increases the production of NF-κB-dependent cytokines [73]. CagA is associated with low-grade chronic inflammation and, as reported by Xia et al. [74], the induction of reactive oxygen species (ROS), which affect the endothelium, and could explain why infection with CagA+ strains of *H. pylori* is more likely to lead to atherosclerosis. Another hypothesis is that anti-CagA antibodies may cross-react with smooth muscle proteins and other cell types responsible for the development of atherosclerosis [75]. Other researchers have demonstrated that CagA antibodies cross-react with two endothelial proteins that have not yet been characterized [76]. Nevertheless, the cross-mimicry between *H. pylori* and host antigens has the potential to trigger the inflammatory processes typically associated with the endothelial layer, contributing to the development of atherosclerotic plaques. In particular, cross-reactivity may occur between antibodies to lipopolysaccharide binding protein (LBP) and antibodies to *H. pylori* heat shock protein 60 (HSP60) and antigens of the endothelium and arterial smooth muscle. In such a scenario, the damage to the endothelium could be mediated by increased activation of the complement protein response [77].

A study by Amedei et al. also highlighted that CagA+ strains of *H. pylori* have a higher capacity to induce IL-6 production [78]. IL-6 is reportedly associated with ageing of both vascular and myeloid cells, which may reinforce each other and promote atherosclerosis [79,80]. Another effect of CagA that likely plays a role in atherosclerosis is its ability to induce macrophage cell formation by downregulating the expression of the transcription factors peroxisome proliferator-activated receptor (PPAR)γ and liver-X receptor (LXR)α [81]. Recently, it has been shown that gastric epithelial cells injected with CagA via the bacterial type IV secretion system, release exosomes containing this protein into the systemic circulation. Exosomes facilitate the transport of CagA into endothelial cells [82]. In a related study, the same research group showed that transgenic mice expressing the CagA on their endothelial cells developed preatherogenic lesions in the aorta when exposed to a high-fat diet. In contrast, non-transgenic mice exposed to the same dietary regimen did not exhibit these changes. Specifically, the aorta of the transgenic mice showed an increase in the thickness of the tunica media and a decrease in its elasticity, which were due to the deposition of extracellular matrix and a decrease in the concentration of elastase in the wall. Furthermore, when the high-fat diet was administered for a longer period, mice with CagA-expressing endothelial cells showed greater macrophage infiltration and development of atherosclerotic plaques [83]. Infection with CagA-positive *H. pylori* strains has been associated with the increased expression of endothelial adhesion molecules such as ICAM-1 and VCAM-1. These molecules are able to bind to circulating monocytes, which facilitates the infiltration of the endothelium by macrophages. The increased expression of adhesion molecules is triggered by the activation of the NLRP3/Caspase-1/IL-1β pathway. Consequently, this activation leads to an increase in IL-6 production, which further promotes local inflammation and contributes, in association with macrophage infiltrate, to the progression of atherosclerosis [84]. Furthermore, in vitro experiments with human endothelial cells expressing CagA showed morphological changes and activation of the proinflammatory transcription factor STAT3, which is involved in the pathogenesis of atherosclerosis [83,85]. The presence of CagA in the vasa vasorum of the aorta in human patients was confirmed by immunohistochemical staining performed on samples obtained after surgical asportation of the aorta [86]. It has been shown that a peculiar polymorphism of IL-1 was related to endothelial injury and to the risk of cardiovascular disease in CagA positive patients [86]. Another group of researchers confirmed these findings. They observed in an animal model that gastric infection with CagA-positive *H. pylori* strains led to increased susceptibility to intimal thickening in the arteries, which was associated with the release of CagA-containing exosomes into the bloodstream. Remarkably, the damage that these exosomes caused to the aortic wall appeared to be caused by the generation of ROS [74]. Shi et al. observed that *H. pylori* was most important in individuals younger than 60 years and without other risk factors. Specifically, *H. pylori* infection was associated with increased carotid intima-media thickness, particularly in CagA+ strains [87]. Consequently, we can establish a link between these studies and conclude that activation of the STAT3 system by CagA at the endothelial level triggers an inflammatory response with the production of ROS. The ROS in turn damage the arterial walls, attract macrophage infiltrates, and contribute to the deposition of fat, eventually leading to the formation of atherosclerotic plaques [85]. It was also found that the vesicles of the outer membrane of the bacterium can transport pathogenic factors to the endothelium and thus promote the development of atherosclerosis [88].

### 4.2. H. pylori, Inflammation and Hypercholesterolemia

There are also other mechanisms linking atherosclerosis and *H. pylori* infection, among which hypercholesterolaemia is one of the most significant. Numerous studies have shown that levels of low-density lipoprotein (LDL) and plasma cholesterol are increased in individuals with *H. pylori* infection. This increase can be attributed to the affinity between CagA and the LDL receptor (RLDL). The spread of CagA in the human body via exosomes from the stomach could potentially cause it to bind to RLDL, preventing LDL cholesterol from being effectively taken up by cells. This interference with the uptake of LDL cholesterol from the bloodstream can lead to hypercholesterolaemia, which is an important risk factor for atherosclerosis and cardiovascular disease [89]. On the contrary, *H. pylori* gastritis has been associated with a reduction in high density lipoprotein (HDL) levels, which is a protective factor against the occurrence of cardiovascular disease and atherosclerosis [90].

Foam cells are essential for the initiation of the atherosclerotic process [91]. *H. pylori* also induces other inflammatory responses in the host that are not always associated with specific components of the bacterium but are nonetheless critical in promoting the inflammatory milieu that links atherosclerosis and *H. pylori* infection. Patients with *H. pylori* infection have higher levels of IL-18. IL-18 is a proinflammatory cytokine that can modulate IFN-γ production and NK cell activity while upregulating FasL expression. These various functions have previously been linked to endothelial dysfunction associated with inflammatory kidney disease [92]. IL-18 is also part of the complex pathway involving the NLRP3 inflammasome and pyroptosis. Pyroptosis, a programmed cell death promoted by caspases, is activated by the NLRP3 inflammasome in response to oxidative stress and ROS and determines downstream production of inflammatory proteins, such as IL-18. In an experiment described by Wu et al. [93], inhibition of this pathway was found to significantly reduce the risk of atherosclerosis in smokers, highlighting its importance in the pathogenesis of the disease. The interaction between NLRP3 and *H. pylori* also involves the production of ROS. It has been observed that *H. pylori* infection is associated with an increase in the levels of ROS through the activation of NADPH oxidase in an inflammation-independent manner. The increase in levels of ROS is associated with activation of NF-κB pathway and interferes with the activity of the PI3K/Akt pathway, a key component in the regulation of important cellular activities [94]. As mentioned above, *H. pylori* can promote a change in the composition of the microbiota (dysbiosis), leading to inflammation of the endothelium and atherosclerosis [95]. While the inflammatory pathway that *H. pylori* can directly activate offers an interesting explanation for how it may determine the onset of atherosclerosis, it is worth noting that modulation of the microbiota may act as another factor in pathogenesis. Specifically, *H. pylori* infection modulates the production of gastrokine (GKN)-1, a protein involved in mucosal repair and healing. Patients infected with *H. pylori* have lower levels of GKN-1, which may not only impair intestinal mucosal healing and promote the development of leaky gut, but also directly affect intestinal eubiosis [96]. Interestingly, the effects of *H. pylori* seem to mainly involve Firmicutes that can produce TMA and TMAO, which are associated with endothelial dysfunction and atherosclerosis [97].

### 4.3. H. pylori: Interaction with Platelets Aggregation

Inflammation is not the only mechanism by which *H. pylori* may promote atherosclerosis. As described by Takeuchi et al. [98], *H. pylori* infection is capable of activating platelets, although the mechanisms are not completely clear. One hypothesis suggests that *H. pylori* may promote the interaction between von Willebrand factor and platelet surface glycoproteins Ib/IX through anti-*H. pylori* IgG and IgG receptors (FcgRIIA) [99]. Another theory is that Lpp20, a lipoprotein bound to the outer membrane of the bacterium, can form immune complexes with platelets to which the host immune system can respond and determine platelet activation [100]. Lpp20 is normally found in extracellular vesicles, which have been linked to the pathogenesis of atherosclerosis in *H. pylori* infections. An additional mechanism that may be responsible for platelet activation by *H. pylori* is proposed based on a study conducted in rabbits. This study highlights that platelet activation and aggregation triggered by *H. pylori* ureases (specifically urease A and B) occurs via a different mechanism that is not initiated by platelet aggregation factor (PAF). Instead, it involves the activation of 12-lipoxygenase and L-type calcium channels [101].

### 4.4. H. pylori and Other Mechanisms Affecting Atherosclerosis

*H. pylori* has also been associated with alterations in lipid metabolism. In a study by Wang et al., eradication was found to improve the lipid profile of patients with dyslipidemia [102]. *H. pylori* also significantly affects HDL levels, and HDL levels increase after eradication [103]. Finally, homocysteine has also been investigated as a possible agent through which *H. pylori* may be able to promote atherosclerosis. It has indeed been observed that *H. pylori* infection can promote hyperomocysteinaemia, a known risk factor in the development of atherosclerosis [104]. Yet, as some authors suggest, there is no available evidence confirming the relationship between these two events [105]. A summary of the different mechanisms linking atherosclerosis and *H. pylori* is reported in Table 1.

## 5. Future Perspective

The importance of the association between *H. pylori* and atherosclerotic disease might lead to the hypothesis that eradication could improve patient prognosis, but results are inconsistent. While eradication should theoretically provide metabolic health benefits [107], some studies have shown that eradication is associated with lower mortality and improved CVD outcomes in younger patients. In patients over 65 years of age, these effects have not been as clear [74]. In a study by Aydemir et al. [108], on the other hand, it appears that eradication positively affects the synthesis of nitric oxide, which is an important regulator of vascular tone, and it could have a positive effect on the progression of atherosclerosis. Another interesting study by Iwai et al. [109] shows another positive effect after eradication. The authors found that in patients undergoing eradication therapy, HDL cholesterol increased significantly, while LDL, platelets, and leukocytes were reduced. The LDL/HDL ratio, which is an important marker of atherosclerosis risk, was thus significantly reduced. Similar results were also observed by Kanbay et al. [110], who also found a decrease in C-reactive protein in patients after *H. pylori* eradication. Eradication also improves overall endothelial health. In patients who had hypertension and were infected with *H. pylori*, there was a decrease in blood pressure and an overall improvement in endothelial function in those who responded to antibiotic therapy [111]. One aspect that must be considered when discussing the potential benefits of eradication therapy is the high incidence of antibiotic-resistant *H. pylori* strains in the general population [112]. In a recent review of the literature, Nista et al. [113] observed for instance that in Italy the rate of antibiotic resistance is so high to even be worth considering clarithromycin quadruple therapy as a first-line treatment [113]. Antibiotic resistance is obviously a problem because of the difficulties it creates to eradicate infection [114], but also because patients have to then undergo multiple therapy lines, which creates problems in itself [115]. In particular, antibiotics have a disruptive effect on the GM, which, as discussed above, plays a role in the development of atherosclerosis [116].

## 6. Conclusions

We described several mechanisms connecting *H. pylori* and the gut microbiota to atherosclerosis. Based on the recent literature, it’s highly likely that *H. pylori* infection plays a role in the development of atherosclerosis, either directly or indirectly by influencing known risk factors. The conflicting results from clinical studies on the impact of eradicating the infection on cardiovascular risk may be due to the adverse effects of antibiotic treatments on the gut microbiota that can contribute to development of cardiovascular disease. Therefore, further studies are needed to investigate not only *H. pylori* eradication but also the potential intricate consequences of eradication therapy on atherosclerosis and cardiovascular diseases.

## Figures and Tables

**Table 1:** *H. pylori* and atherosclerosis: overview of pathogenic mechanisms.

Pathogenic Pathway	Effect	Model	Reference
LPS	Th1/Th2-activation Low grade inflammation through DCSIGN/lectin pathway	Murine	[69]
HP-NAP	IL-8, IL-12, IL-23 ↑ MIP-1α, and MIP-1 ↑ Mast cell activation Increased IL-6 and TNF-α	In vitro Murine	[70]
VacA	TNF-α, MIP-1α, IL-1, IL-6, IL-10, and IL-1 ↑ Inhibited differentiation of T lymphocytes	In vitro Murine Human	[71]
CagA	Autophagy in host cells ↓ IL-1α, IL-6, IL-8, and IL-18 ↑ c-Met activation PI3K/AKT/mTOR pathway activation Accumulation of SQSTM1 ROS ↑ Cross-reactivity PPARγ and LXRα ↑ Foam cells ↑	In vitro Murine Human	[73,74,75,78,79,80,81,91]
NLRP3	Pyroptosis ↑ ROS ↑ Activation NADPH oxidase NF-κB pathway activation PI3K/Akt pathway activation	In vitro Murine	[94]
GKN-1	Microbiota modulation Leaky gut Firmicutes↑ TMA/TMAO↑	Human	[96]
Platelet activation	Interaction between von Willebrand factor and platelet surface glycoproteins Ib/IX ↑ 12-lipoxigenase pathway	Rabbit Human	[100,101]
Lpp20	Platelet immune complexes	Human	[100]
Extracellular vesicles Outer membrane vesicles	Transport of pathogenic factors	Murine Human	[88,106]
Lipid metabolism	HDL ↑ after eradication	In vitro Murine Human	[103]

## Data Availability

Not applicable.
